# A systematic review of steroid use in peripheral nerve pathologies and treatment

**DOI:** 10.3389/fneur.2024.1434429

**Published:** 2024-09-02

**Authors:** Brandon Couch, Dan Hayward, Gracie Baum, Naveen Arunachalam Sakthiyendran, Justin Harder, Evan J. Hernandez, Brendan MacKay

**Affiliations:** ^1^Texas Tech University Health Sciences Center, Lubbock, TX, United States; ^2^Department of Orthopaedic Hand Surgery, Texas Tech University Health Sciences Center, Lubbock, TX, United States

**Keywords:** corticosteroid treatment, peripheral nerve damage, peripheral neuropathy, neuropathic pain, steroid nerve block

## Abstract

**Background:**

The use of corticosteroids has become a part of the standard of care in various pathologies but their use in peripheral nerve injury treatment is limited. Given corticosteroids’ anti-inflammatory properties and their regulatory role in neuronal protein production and myelination, corticosteroids could serve as an adjunct therapy for peripheral nerve injuries. This review aims to systematically investigate the current use of corticosteroid treatment in peripheral nerve pathologies.

**Methods:**

The systematic search was performed on PubMed, MEDLINE, EMBASE, Scopus, Cochrane, and Web of Science using keywords such as “corticosteroid treatment,” “peripheral nerve damage,” “peripheral neuropathy,” and “complications.” The PRISMA guidelines were used to conduct the systematic review and all articles were reviewed by the corresponding author. After the initial search, individual study titles and abstracts were further screened and categorized using an inclusion and exclusion criteria followed by a final full-text review.

**Results:**

Out of the total 27,922 identified records, 203 studies were included based on the selection criteria. These studies focused on the use and efficacy of steroids across a spectrum of compression and non-compression peripheral neuropathies such as cubital tunnel syndrome and chronic inflammatory demyelinating polyradiculoneuropathy. Various studies noted the promising role of steroids in offering pain relief, nerve block, and nerve regeneration effects. Additionally, safety considerations and potential complications regarding steroid use in peripheral nerve injuries were analyzed.

**Conclusion:**

While there is currently limited clinical utilization of corticosteroids in peripheral nerve pathologies, the anti-inflammatory and regenerative effects that steroids provide may be a beneficial tool in managing various peripheral neuropathies and their associated pain. Additional clinical trials and investigation into the mechanism of action could improve the reputation of steroid use as peripheral nerve injury treatment.

## 1 Introduction

Corticosteroids, such as hydrocortisone, dexamethasone and prednisone, are among the most widely prescribed drug classes worldwide (1). They are used for numerous conditions including inflammatory disorders, allergic and autoimmune reactions, neurological disorders, prevention of graft rejection, and shock. Steroids are also considered the non-operative standard of care for carpal tunnel syndrome (CTS) (2–5), but there is a lack of agreement regarding the use of steroids in other peripheral nerve disorders.

Under normal physiological conditions, production of proteins within the neuron is partially regulated by corticosteroids. Within the cell body, steroid receptors are activated, dimerized, and eventually influence gene expression leading to protein production modulation. One such receptor is present within Schwann cells, and the binding of glucocorticoid steroids activates myelin associated protein synthesis, specifically glycoprotein Po and peripheral myelin protein 22 (6–12). Synthesis of these myelin proteins has also shown to have regenerative effects on damaged peripheral nerves. Therefore, steroids could be a beneficial means of tissue regeneration by enhanced Schwann cell myelin sheath protein synthesis (13, 14).

Following nerve injury, the primary barriers for axonal nerve regeneration are excessive inflammation and a lack of remyelination by Schwann cells (15, 16). Glucocorticoid steroid treatment can potentially address both aforementioned barriers. Nerve damage from either trauma, surgery, or neuropathies can result in neuropathic pain, which has been linked to pro-inflammatory states, mediated by bradykinin, interleukins 1,6, and 8, along with tumor necrosis factor (TNF) and C-reactive protein (17). Corticosteroids are effective at this level of pain attenuation due to their anti-inflammatory properties (18, 19). The mechanism of attenuation involves peripheral inhibition of phospholipase, which has a down-stream effect of reduced cyclooxygenase and lipoxygenase pain-aggravating products (20).

Despite evidence that steroids increase myelination and attenuate inflammation in damaged nerves, there is not a widely accepted treatment algorithm for use of steroids in various peripheral nerve conditions. This review illustrates a lack of cohesive literature examining the use of these therapeutic strategies in peripheral nerve pathologies and highlights that, in certain cases, steroids can serve as a valuable adjunct in multi-modal pain treatment.

## 2 Methods

### 2.1 Search terms and strategy

A PRISMA review of the following databases was conducted: PubMed, MEDLINE, EMBASE, Scopus, Cochrane, and Web of Science, using keywords: ((((((((((((((((((“peripheral nerve” or “peripheral neuropathy”) AND (“injury” or “regeneration” or “myelination”)) OR (steroids peripheral myelin protein)) OR (steroid nerve block)) OR ((“neuropathic pain” or “peripheral neuropathy” or “nerve pain” or “peripheral nervous system” or “peripheral nerve damage”) AND (“corticosteroid” or “steroid”))) OR ((“corticosteroid treatment” or “steroid treatment” or “dexamethasone” or “methylprednisolone” or “prednisolone”) AND (“peripheral nerve”) AND (“safety” or “adverse effects” or “side effects” or “complications”))) OR ((“corticosteroid treatment” or “steroid treatment” or “dexamethasone” or “methylprednisolone” or “prednisolone”) AND “peripheral nerve”)) OR ((“median nerve entrapment” or “carpal tunnel syndrome” or “pronator syndrome”) AND (“treatment” or “steroid injections” or “oral steroid” or “dexamethasone” or “methylprednisolone” or “prednisolone”))) OR ((“ulnar neuropathy” or “ulnar neuritis” or “ulnar nerve entrapment” or “cubital tunnel”) AND (“treatment” or “steroid”))) OR ((“brachial neuritis” or “parsonage-turner syndrome”) AND (“treatment” or “steroid”))) OR ((“ulnar neuropathy” or “ulnar neuritis” or “ulnar nerve entrapment” or “cubital tunnel”) AND (“treatment” or “steroid”))) OR ((“nerve compression syndrome” or “nerve entrapment”) AND (“treatment” or “steroid”))) OR ((“radial neuropathy” or “radial nerve entrapment” or “radial tunnel”) AND (“treatment” or “steroid”))) OR ((“meralgia paresthetica” or “femoral neuropathy”) AND (“treatment” or “steroid”))) OR (“peripheral diabetic neuropathy” AND (“treatment” or “steroid”))) OR (“chemotherapy-induced peripheral neuropathy” AND (“treatment” or “steroid”))) OR (“alcoholic peripheral neuropathy” AND (“treatment” or “steroid”))) OR (“chronic inflammatory demyelinating polyradiculoneuropathy” AND (“treatment” or “steroid”))) OR (“leprous neuropathy” AND (“treatment” or “steroid”)).

Two reviewers (B.C., D.H.) reviewed the studies independently and any inconsistencies between reviewers were resolved by the corresponding author (B.M.). The articles were screened for inclusion by title and abstract, then by full text to assess for eligibility. Our search was limited to English language articles (or those with available English translations) published from January 1975 through June of 2022. The literature search focused particularly on evidence-based data regarding the mechanism of action of corticosteroids on healthy and pathologic nerves, and the clinical utility of steroids for treatment of various peripheral nerve conditions.

### 2.2 Eligibility criteria

The inclusion criteria for articles screened for eligibility were: (1) all studies involved corticosteroid use as a treatment modality; (2) included patients that were treated for peripheral nerve pathologies; and (3) English language articles only. Studies were excluded if they met the following criteria: (1) no clinical or translational component; and (2) were not available for full-text viewing.

### 2.3 Data extraction and main outcomes

Following screening and selection of articles, the data was extracted using a standardized format (Microsoft Office Excel 2024). After all articles were selected, it was collectively decided by the authors to collate the data into different subsections including: oral steroids as pain relief, nerve block, and nerve regeneration, and corticosteroid use in various compression and non-compression neuropathies and associated side effects.

## 3 Results

**Figure 1:**
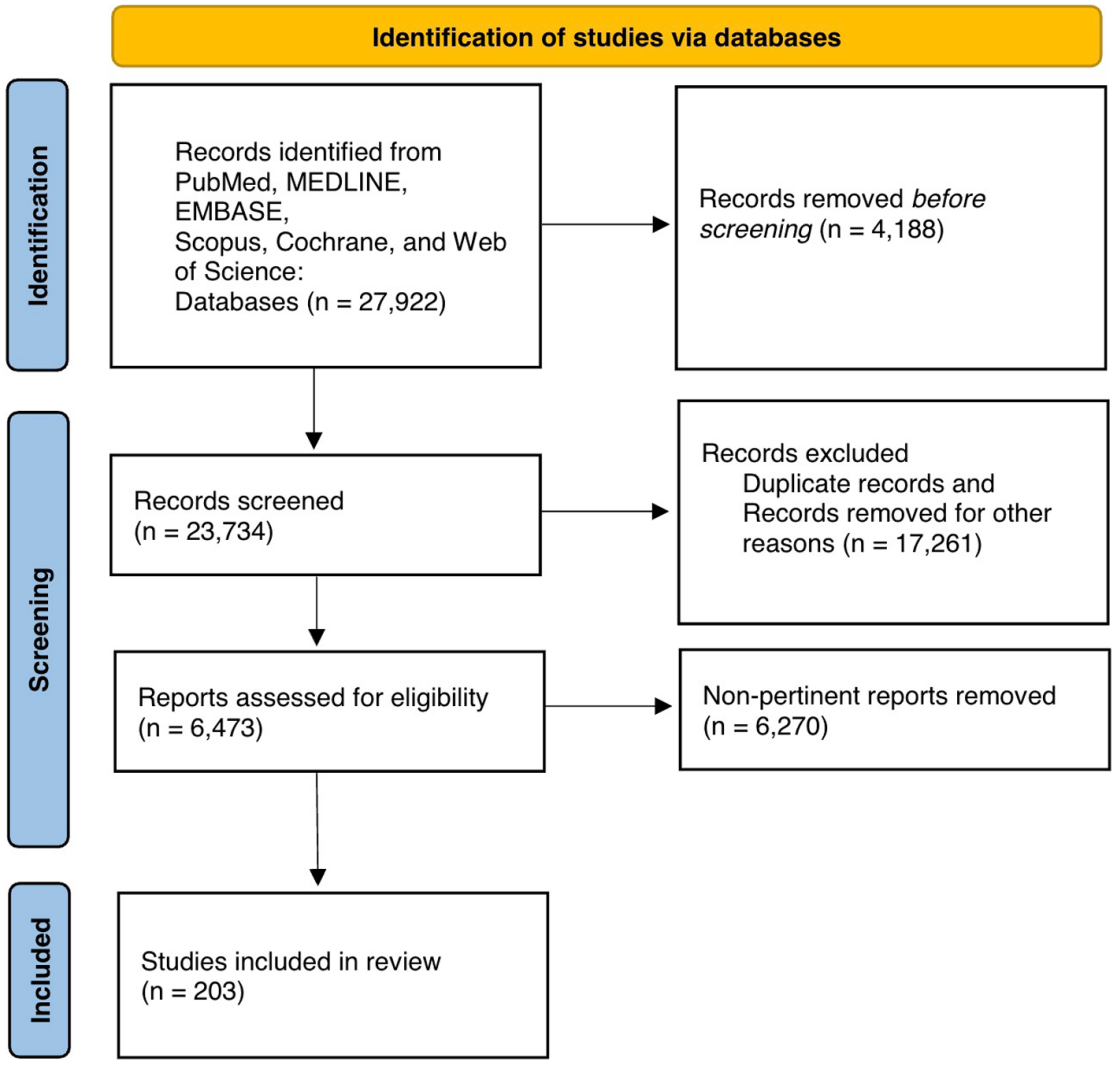
PRISMA flowchart.

Figure 1 exhibits the PRISMA flow diagram representing article retrieval and screening. There were 27,922 records identified, of which 23,734 records underwent further screening. A total of 6,473 records were identified after excluding duplicate articles and these records underwent full-text review. After screening, 203 articles met inclusion criteria for our synthesis of the literature in this systematic review (Figure 1). Included papers were then divided into different categories of steroid use and peripheral neuropathies and the content of these manuscript texts were analyzed.

## 4 Discussion

### 4.1 Oral steroids as pain relief adjuvant

Tissue injury triggers the release of proinflammatory cytokines and prostaglandins (20). Cytokines and prostaglandins elicit a pain response through inflammatory and neuropathic mechanisms. Topical TNF-alpha application can induce spontaneous depolarization of nociceptive neurons, and prostaglandins are linked to enhanced pain response after neuronal production (21).

The ubiquitous expression of steroid hormone receptors in the PNS suggest their role is pivotal in pain perception via neuronal maturation, differentiation, and plasticity (22). Accordingly, glucocorticoids inhibit phospholipase-A2 metabolism of membrane phospholipids to arachidonic acid therefore decreasing production of inflammatory cytokines (23). From this primary mechanism of action, a reduction of circulating TNF-alpha and prostaglandins may correspondingly diminish spontaneous discharge from damaged nerves therefore blunting the nociception pathway. This effect has also been demonstrated in pre-clinical models. Hargreaves et al. found that dexamethasone administration (125 mg dosage preoperatively) in rats decreased tissue levels of bradykinin, a proinflammatory peptide (24). Takeda et al. reduced mechanical allodynia and thermal hyperalgesia in mice through methylprednisolone administration (dosage at 4 mg/(kg*day) infusion systemically, or 80 micrograms/(kg*day) intrathecally) following spinal nerve ligation (25). The reduction in cytokine-induced nerve firing could therefore be responsible for the reduction in pain.

The effect of perioperative glucocorticoid administration for postoperative pain scores has been well studied with consistent results. Preoperative methylprednisolone decreased postoperative inflammatory cytokines, IL-6, TNF-alpha, and E selectin, levels after cardiopulmonary bypass surgery (26). A systematic review of perioperative dexamethasone administration lowered pain scores recorded 2 hours after surgery with minimal adverse side effects (27). This decrease in perceived postoperative pain is further illuminated by the quantity of pain medications required to achieve acceptable postoperative pain levels. Traditionally, postoperative pain is controlled with opioids; however, opioids have well known addictive and harmful properties. Patients receiving glucocorticoid treatment require fewer oral opiates following surgery (20, 27, 28). A 2012 systematic review and meta-analysis reported that patient treated with dexamethasone used less opioids at 2 h and 24 h after surgery. The nine studies (978 patients) recording opioid use 2 h post-operatively demonstrated a 13.0% decrease in pooled opioid consumption compared with control, and the 14 studies (2,157 patients) recording use at 24 h demonstrated a 10.3% reduction (27).

### 4.2 Nerve block

Steroid use is also efficacious for palliation of post-operative pain when combined with an analgesic block for suppression of nociceptive pathways. Multiple systematic reviews examining randomized controlled trials have concluded that duration of analgesia in peripheral nerve blocks can be significantly increased by the addition of perineural dexamethasone compared to standard treatment (29, 30) with some meta-analyses reporting an additional 5 h or more of analgesia time (31, 32). It is additionally possible that the steroid dose could influence analgesia length. In one study, ultrasound-guided perineural dexamethasone injection of 1, 2, 3, and 4 mg’s alongside ropivacaine for brachial plexus nerve block extended analgesia time by 835, 904, 965, and 1,023 min, respectively (33). However, dexamethasone may have a ceiling dose for prolonged pain control. Two systematic reviews concluded that there was no evidence to support dose quantities above 4 mg of dexamethasone, specifically finding 4 mg to be just as effective as 8 mg doses (34, 35).

Additionally, dexamethasone has been shown to increase duration of analgesia compared to dexmedetomidine, an alpha-2 adrenergic receptor agonist (36, 37). Although these reviews have exclusively examined dexamethasone, methylprednisolone has also shown promising results as a nerve block adjuvant. Eker et al. treated patients with postinjury neuropathic pain symptoms by injecting 0.5% lidocaine solutions with 80 mg depo-methylprednisolone via peripheral nerve blocks at the proximal site of the injury, resulting in improved pain score outcomes at 3 months compared to injecting 0.55 lidocaine alone. These results were attributed to the reduction of proinflammatory cytokines and prevention of spontaneous nociceptive neuronal firing (38).

These studies suggest that corticosteroids have analgesic effects and can be effective therapy for neuropathic pain due to nerve injury by decreasing the production of local inflammatory mediators and ectopic neuronal discharge at the nerve injury site. Thus, patients who develop uncomfortable dysesthesia post-operatively may be good candidates for steroid treatment, as the benefits of dysesthesia reduction often outweigh the risks associated with corticosteroid use.

### 4.3 Nerve regeneration

The use of local steroid injection for peripheral nerve regeneration therapy in humans is limited to small distances, and molecular nerve regeneration therapy is still primitive. Specifically, human axonal regeneration occurs at a rate of about 1–2 mm per day, and the majority of steroid nerve regeneration research exists in animal studies (39). Current literature shows no established adjuvants that accelerate peripheral nerve regeneration (40).

Decreased muscle innervation from peripheral nerve injuries may result in atrophy and loss of function. This element of peripheral nerve injury pathophysiology could potentially be addressed by steroid treatment. Two rat-model studies recorded hypertrophy of previously atrophied muscle from sciatic nerve injury after treatment with steroids (dosed at 1–2 mg/Kg for 1–28 days postoperatively) (41, 42). These effects were attributed to nerve regeneration with resulting increased innervation.

Similar studies found that following crush injury of the sciatic nerve, function in the rats’ hindlimbs were improved after treatment dexamethasone, methylprednisolone, or betamethasone. The functional improvement was associated with more pronounced remyelination, decreased inflammatory cell infiltrate in surrounding tissues (43–46), and Schwann cell proliferation (47–49). The health of the myelin sheath corresponded with the strength of the muscle innervation which directly resulted in increased functionality and size of the previously damaged myotomes. Additionally, facial and median nerve function studies found significantly improved myelin sheath thickening and functional recovery after glucocorticoid treatment (at a 5 mg/mL dosage) compared to controls (50, 51). While most studies utilized local steroid injection, topical dexamethasone (dosage of 0.1 mg/kg) has also shown nerve health promotion in rat models after crush injuries (52–54).

The rat model’s greatest limitation is the short length of the sciatic nerve, making large-gap regeneration difficult to assess. Nevertheless, these studies still provide a foundation for additional research. The first case report for successful post-traumatic use of corticosteroids in humans was published in 2020, in which three local peri-neural injections of 40 mg methylprednisolone returned the patient to a normal ulnar nerve function (55). Following 8 years of complete sensory and motor loss of the right ulnar nerve, this patient regained function in both categories following local injection (55). This case report demonstrates promising recovery of nerve function following non-surgical, local steroid injection-based treatment. Additionally, a retrospective study investigating the use of “pulsed” intravenous methylprednisolone treatment (at a dose of 1 g/day) on neuritis indicated that this form of non-oral steroid treatment was also an effective modality in preserving nerve function (56).

### 4.4 Compression neuropathies

Neuritis of the upper extremity commonly manifests as CTS along the median nerve, and cubital tunnel syndrome (CuTS) along the ulnar nerve. Although CTS and CuTS are the most prevalent compression neuropathies, any nerve may be compressed at any point along its course. Other common entrapments include the ulnar nerve in Guyon canal syndrome, pronator teres can entrapment of the median nerve or anterior interosseous nerve by the pronator teres muscle in the proximal forearm (57–59), posterior interosseous nerve compression by the supinator muscle, and the superficial radial nerve compression in Wartenberg’s syndrome (59, 60).

Compression syndromes are often the result of external mechanical pressure (e.g., medical equipment), anatomical factors (e.g., cysts), or local inflammatory processes (e.g., arthritis) (61). Upper extremity entrapment neuropathies have traditionally been treated by surgical means; however, nonsurgical treatment modalities including ultrasound guided steroid injection have revealed a possible alternative for standard of care (60, 62, 63). While there have been prospective trials revealing propitious results for conservative steroid treatment (6 mg/mL of celestone) for nerve entrapment, there are still a lack of prospective randomized controlled studies to evaluate these claims (64–66).

#### 4.4.1 Carpal tunnel syndrome

The use of injectable and oral steroids for treatment of carpal tunnel syndrome (CTS) has been extensively researched with primarily favorable results. The articles included in this review observed the effects of treatment in a total of 3,641 patients. Pain mitigation effects of local glucocorticoid steroid injections (average dose of ~40 mg) for treatment of CTS are most notable in the short-term (67–69). Marshall et al. found improved clinical symptoms of CTS following steroid injection at 1 month follow-up visits; this effect lacked statistical significance beyond 1 month (70). Other studies found similar results in short-term intervals but could not differentiate from the control within a year of the injection (71, 72). Despite the limited, short-term alleviatory effects of steroid injections for CTS, Atroshi et al. noted that methylprednisolone injections (dosed at 80 mg or 40 mg), reduced the rate of surgery 1 year following treatment (73). However, surgery becomes necessary in long-term management of most patients (74).

The effectiveness of steroid injections for treatment of CTS has been quantitatively illustrated by sensory and motor nerve conduction studies (NCS). Local steroid injections improved motor and sensory NCS values in over 60% of CTS cases examined (75, 76). The positive NCS effects are present in both the short-term and long-term, over 6 months (77, 78). This favorable conduction effect was expounded by Cartwright et al. using ultrasound to reveal increased cross-sectional area and vascularity of the median nerve after local steroid injection which directly correlated with CTS symptom scores. In addition, Stepic et al. found that intraoperative local steroid injection improved NCS values as compared to surgical release alone (79). However, Mottaghi et al. found no significant difference between intraoperative steroid injection and carpal tunnel release alone (80).

Oral steroids have also been utilized for alleviation of CTS symptoms. Most studies have found similar results using oral steroids as they have found with local steroids: improved symptoms in the short-term, but significant differences wane over time (81–84). At two-week follow-up visits, both oral and local steroids provided similar relief; however, by one-month, only local steroid injections exhibited a positive significant difference in alleviation of CTS symptoms (70, 85, 86).

#### 4.4.2 Cubital tunnel syndrome

CuTS is the second most common upper extremity neuropathy (87), and surgical treatment is currently the treatment of choice (88, 89). Filippi et al. concluded that simple decompression of the ulnar nerve is successful treatment for CuTS as only three of forty surgically treated patients lacked improvement following the procedure (90). However, surgery always has associated risks and ulnar nerve transposition can decrease blood flow and possibly kink the nerve, requiring additional surgical correction (91). Due to this, nonsurgical methods for treatment of CuTS are being pursued. Common approaches include activity modification, splinting, steroid injection, and physical therapy (92–94). However, systematic reviews of conservative cubital tunnel management have shown limited evidence-based literature to guide conservative treatment with most studies lacking controls and long-term patient follow-ups (95, 96).

Some research has found potential improved clinical outcomes by conservative steroid treatment; however, these findings are not consistent. A study of 10 patients examined neuronal regeneration effects of ultrasound-guided triamcinolone injection (dosed at 40 mg) for CuTS and found significantly improved conduction velocity and cross-sectional area (97). These results are further supported by a case series of 63 patients which found ultrasound guided corticosteroid injections (methylprednisolone acetate dosed at 40 mg) to provide transient relief (98). Stutz et al. reported an improvement in disability, as indicated by decreased DASH scores in the nonsurgical treatment group; however, the improvement was less than that seen in the surgically treated group (99). Two small studies found that steroid injection for ulnar neuropathy resolved symptoms in 4 of 7 and 5 of 8; however, they acknowledged the limitations of their studies’ small sample sizes and suggested the need for further examination (94, 100). In a systematic review, Kooner et al. found limited benefit of steroid injection for cubital tunnel syndrome compared to the other modalities (95). Specifically, one study of 55 patients found no difference in outcome between steroid treatment and placebo while another small case series with 10 patients, found no difference between steroid (1 mL of methylprednisolone acetate dosed at 40 mg) and splinting (101, 102). These results mirrored another study of 36 patients where comparison of corticosteroid injection to dextrose injection yielded no significant difference (103). Specifically regarding Guyon canal compression, Earp et al. concluded that nonsurgical treatment is successful in entrapment resulting from excessive, repetitive use (104). While multiple studies have reported improved outcome with steroid treatment in CuTS (94, 97–100), other studies have refuted these claims (95, 101, 102). Given the paucity and heterogeneity of published data, more robust future research should be conducted and may show different results.

#### 4.4.3 Radial nerve compression syndromes

Similar to CuTS, there is a general lack of controlled studies comparing steroid use versus surgery for treatment of radial nerve compression syndromes. The radial nerve may become compressed between the brachioradialis and extensor carpi radialis longus proximal to the wrist, at the radial tunnel distal to the elbow, and after mid-humerus fractures (104). Surgical release of the supinator muscle for radial tunnel syndrome is commonly successful and indicated when conservative treatment fails (105). Despite the success of surgical decompression, conservative treatment should be attempted first due to risks accompanying surgery and loss of function due to muscle attachment release. Auspiciously, nonsurgical corticosteroid therapy has been successful in clinical studies for radial tunnel syndrome. A study of 40 patients reported a significant decrease in DASH scores of patients treated with a single betamethasone injection at the origin of the posterior interosseous nerve, while another study of 54 patients reported only 2% of patients retained pain after infiltration of a local anesthetic and betamethasone solution (dosed at 0.75 mL of 6 mg/mL Celestone) (64, 65). Additionally, symptom relief by corticosteroid injection is a determinant of the often-confounding clinical diagnosis of radial tunnel syndrome (106).

#### 4.4.4 Pronator syndrome and anterior interosseus nerve syndrome

Median nerve compression, although most commonly occurring as CTS, can arise at the proximal forearm due to pronator entrapment as pronator syndrome (PS) or anterior interosseous nerve syndrome (AIN). PS presents similarly to CTS and is marked by vague pain, paresthesia, and limited motor defects; contrariwise, AIN is exclusively a motor palsy (107–109). Both surgical and nonsurgical treatment options are used in these syndromes, but, unlike CuTS, surgical intervention is only indicated when nonsurgical treatment fails (108–110). This treatment algorithm is largely due to the success of noninvasive treatment. Conservative treatment includes rest, activity modification, splinting, physical therapy, and corticosteroid injections (110). Although a multi-model treatment regimen should be utilized, corticosteroid therapy has been shown to be beneficial on its own. Delzell et al. treated 14 patients with ultrasound-guided perineural hydrodissection and corticosteroid injection (0.5 mL of dexamethosone sodium phosphate dosed at 4 mg/mL) and found significant relief in 70% of those treated (111). Corticosteroid injection is a prominent component of treatment for the median nerve compression syndromes (CTS, PS, and AIN) and has an increasing presence in the alleviation of other upper extremity peripheral neuropathies.

#### 4.4.5 Meralgia paresthetica

Meralgia paresthetica, a common lower extremity compression syndrome, is a mononeuropathy of the lateral femoral cutaneous nerve. Although this syndrome can be caused by a multitude of factors, it has been linked to pregnancy especially when women maintain a prolonged lithotomy position (112). Like many other compression syndromes, meralgia paresthetica results in pain and paresthesia. Analysis of nonsurgical treatment using corticosteroids shows primarily positive results. Two studies examined the effects of corticosteroid perineural injection in 20 patient sample sizes each. One study that used 1 mL of methylprednisolone acetate (40 mg/mL) reported decreased symptoms in 80% of patients by the first week, and in 100% by 2 months following methylprednisolone injection. The second study found complete and partial resolution of symptoms in 75% and the remaining 25% of patients, respectively, with triamcinolone injection (1 mL dosed at 10 mg/mL) (113, 114). Kloosterziel et al. treated 10 patients with 1 mL of methylprednisolone-lidocaine solution injection and 10 with saline as a placebo and found a significant reduction in pain in the placebo group but not the treated group (115). These results were perplexing and likely attributed to the small sample size. A larger study of 54 patients compared ultrasound-guided betamethasone injection to a TENS group and mock TENS control and observed a statistically significant decrease in pain in the corticosteroid treated group as compared to the others (116). Even though corticosteroid injections provide symptom improvement in a majority of patients, surgical decompression remains necessary as a final option when others fail. Surgical intervention appears highly successful with two studies reporting long-term relief of symptoms in 100% of patients who corticosteroid treatment was unresponsive and surgical management was required (117, 118). Another report found improved symptoms in 93% of surgical cases and noted that obese patients were 6 times more likely to have persistent symptoms after surgery (119). The current data shows a compelling argument for ultrasound-guided perineural corticosteroid injection. Nonetheless, the lack of large sample size, prospective, controlled studies examining treatment options for meralgia paresthetica makes deciding upon a comprehensive regimen difficult.

### 4.5 Non-compression neuropathies

Neuropathy is possible without compression from an outside source. Non-compression neuropathies are often complex pathologies, rising from metabolic, immune-mediated, and idiopathic sources. Metabolic and immune-mediated neuropathy management involves treatment of the underlying pathology, as well as the nerve itself. Peripheral steroid injection data is scarce for these pathologies, creating opportunity for future investigation.

#### 4.5.1 Metabolic neuropathies

Diabetic peripheral neuropathy is a common sequela of diabetes mellitus, occurring in 25% of diabetic patients (120). This condition is likely caused by microvasculitis and can present as burning pain, paresthesia, and weakness (121, 122). Although pregabalin and duloxetine are the only drugs approved by the US FDA, prior research has indicated that corticosteroid treatment may be effective for treatment of diabetic peripheral neuropathy (121, 122). Pulsed oral prednisolone and intravenous methylprednisolone treatment have resulted in improved symptoms when administered near the time of symptom onset (123, 124). In the retrospective study, nine patients with diabetic amyotrophy were treated with pulsed oral or intravenous methylprednisolone. It was found that treatment started within 2 months of symptom onset were associated with rapid improvement in pain, and treatment started within 4 weeks of symptom onset resulted in rapid improvement of both strength and pain. Blood glucose increased on treatment days but no patient necessitated lasting changes in diabetic treatment as the result of this therapy and no other serious adverse effects were seen (124). Rat-models have shown advantageous effects of steroids within the nerves of diabetic rats, but there have not been any randomized controlled studies examining these effects in diabetic humans (125, 126). Additional studies may identify generalizable treatment regimens of oral and intravenous steroids for diabetic neuropathy, as well as explore local injection as a route of administration.

Alcoholic neuropathy, the result of chronic excessive alcohol consumption, is another complicated neuropathy with many proposed etiologies and a nonexistent evidence-based therapy. This condition can be severe for patients with the primary symptom being pain (127). Alcohol consumption limitation and cessation are the mainstays of prophylaxis, and diet supplementation (primarily B vitamins) has been attempted with primarily futile results (128). Because there is no clear therapy of choice, presumed contributing factors have been investigated and may provide insight for future treatment. In rat models, chronic ethanol consumption decreases nociceptive thresholds corresponding with increased proliferation and activation of microglia (129). Additionally, they have high levels of protein kinase C (PKC) in dorsal root ganglia, and PKC inhibitors lesson hyperalgesia in these models (130). Therefore, PKC inhibitors and treatment aimed at reducing microglia activity could prove useful in pain attenuation. In humans, excessive alcohol consumption potently activates the hypothalamic–pituitary–adrenal (HPA) axis leading to a sustained sympathetic response which is associated with neuropathic states (131). Prolonged prednisolone administration is known to cause HPA axis suppression (132). Because of this characteristic of alcoholic neuropathy, it is possible that steroid treatment could provide therapeutic effects.

#### 4.5.2 Immune-mediated neuropathies

Inflammatory demyelinating neuropathies, including chronic inflammatory demyelinating polyradiculoneuropathy (CIDP), Guillain-Barré syndrome (GBS), and multifocal motor neuropathy (MMN), are a rare but severe set of nerve disorders. Common symptoms include weakness, sensory loss, inability to walk, and difficulty with activities of daily living (133, 134). Specifically, CIDP is a progressive, relapsing disease characterized by symmetrical weakness progressing over at least 2 months (133, 135). The etiology varies slightly between the disorders but retains a common theme of autoantibodies towards the nodes of Ranvier or elements of myelin within peripheral nerves (136). CIDP is the most common of the immune neuropathies and involves a difficult diagnosis based on clinical characteristics and electrophysiological evidence of demyelination (137). Based on the autoimmune nature of these illnesses, immunosuppression is the general method of treatment. CIDP has been successfully treated with corticosteroids, intravenous Immunoglobin (IVIg), and plasmapheresis (133, 135, 136, 138–141). Although steroids are effective in treatment of CIDP, they have not shown benefit for GBS or MMN, and IVIg is therefore indicated (142–144). In spite of IVIg’s perceived versatility in the treatment of inflammatory demyelinating neuropathies, they are significantly more expensive than alternative treatments and a reason for exploration of corticosteroid utility as treatment (139).

The effect of steroid treatment for CIDP has been examined by non-controlled studies with favorable results. Two trials, one of ten patients and the other of 125 patients, treated patients with pulsed corticosteroids (dosed at 500 mg once a week and adjusted every 3 months) found most patients responded well, with roughly 60% of patients achieving long-term remission (145, 146). These results were similar to two other studies which concluded that long-term remission of CIDP could be achieved by pulsed dexamethasone or prednisolone therapy (dosed at 60 mg/day for 5 weeks and then tapering to zero) (147), and that pulsed-oral corticosteroid therapy is safe and effective for long-term treatment of CIDP in children (148).

Two randomized, controlled trials, one of 40 and the other of 35 patients, reported significant improvement in the corticosteroid-treated group (1 mg/kg) over the control group (149, 150). Yet, steroids appear to be highly effective in some patients while ineffective in others. Determining which patient characteristics are most likely to respond to corticosteroids could increase success rates in the chosen cohorts. Two studies notably observed a significant association between a favorable response to corticosteroid treatment (0.5–0.75 mg/kg/day of prednisone) with shorter disease duration prior to treatment onset, lesser axonal damage or impaired nerve conduction velocity, being female, and being of a younger age (140, 151). These disease-related characteristics should be considered when choosing a therapy regimen.

Although steroids have documented efficacy in treatment of CIDP, it is important to consider if it is truly the best treatment. Many studies have attempted to clarify a treatment of choice for CIDP by comparing the outcomes of patients treated with IVIg and corticosteroids. However, results have done little to differentiate between the two and often complicate the decision. One study reported favorably for steroids stating that steroid response rate was significantly higher than IVIg in patients with normal or moderately enlarged cross sectional area of the nerve; nevertheless, this difference was not found in patients with enlarged cross-sectional area (152). Of note, multiple studies have found no significant difference in patient outcomes or short-term efficacy between the two therapies (133, 153–155), yet other studies have suggested that IVIg is more effective in the short-term while corticosteroids are more effective in the long-term for remission (138, 139). Further confounding the decision, a study of 45 patients found that more patients had to stop intravenous methylprednisolone than IVIg due to adverse effects while more patients on IVIg experienced worsening symptoms after therapy discontinuation than those treated with methylprednisolone (which was dosed at 0.5 g in 250 mL sodium chloride solution for 4 consecutive days) (156). A combination therapy of IVIg and corticosteroids may possibly deliver the preferable results of each: short-term efficacy from IVIg and long-term remission from corticosteroids. Adrichem et al. explored this hypothesis by treating 20 CIDP patients with IVIg and intravenous methylprednisolone (with a cumulative dose of 7 mg) and found the treatment to be well tolerated yielding remission in nearly 60% of patients (157). The results of this study are suggestive of a potential treatment of choice for CIDP, but more extensive investigation is needed.

Ultimately, the choice of treatment should be made with careful analysis of risk to benefit. The complexity and progressive nature of CIDP combined with sustained immunosuppressive therapeutics makes prognosis difficult, and the detrimental effects of each therapy cumulate to increase long-term morbidity (158). Additionally, multiple studies have reported issues with long-term steroid use in CIDP leading to discontinuation of the therapy, notably steroid-induced osteoporosis in the elderly population (145, 146, 156, 159).

Leprous neuropathy, as a result of *Mycobacterium leprae* infection, is commonly treated with steroids. However, systematic reviews have questioned the efficacy of this treatment, finding inadequate evidence to advocate for steroid therapy (101, 160). Two placebo-controlled trials, one of 92 patients and the other of 75 patients, contrasted prednisolone (which was dosed at 40 mg/day, tapred by 5 mg every 2 weeks for a total of 16 weeks) to placebo treatment and reported that there was no significant difference in sensory improvement between the two groups (161, 162). Despite the lack of favorable placebo-controlled evidence, assessing outcomes of short-term and long-term steroid administration for leprous neuropathy has shown benefit for longer treatment. Sundar et al. found patients receiving short-term, 12 weeks, steroids were more likely to require additional steroid treatment for alleviation of symptoms than the group receiving long-term, 20 weeks, steroid treatment (163). This positive effect of prolonged treatment was demonstrated in a 2008 case report of a leprosy patient with complete and partial conduction loss of the ulnar and median nerve, respectively. Monthly dexamethasone injection for 6 months resulted in significantly increased motor conduction velocity and sensory function of the ulnar and median nerves (164). The benefit to prolonged steroid use, however, does seem to have a limit. Wagenaar et al. found no difference in outcome between a 20-week course and 32-week course of prednisolone administration (dosed at either 45 or 60 mg/day based on the patient’s body weight) (165). Steroid use, although favorable for many neuropathies, shows limited evidence supporting its therapeutic effects for treatment of leprous neuropathy.

#### 4.5.3 Cancer treatment related neuropathies

Chemotherapy-induced peripheral neuropathy (CIPN) and post-radiation neuritis are common in cancer pain cases, accounting for approximately one-third of cases (166). The pain mechanism in this setting is poorly understood and often complex, involving neuropathic, inflammatory, and possible ischemic components (167, 168). Furthermore, CIPN may vary from patient to patient, tumor to tumor, and site to site (169). CIPN occurs in 30–40% of patients treated with neurotoxic chemotherapy agents, including Cisplatin, taxanes, and Bortezomib (170). CIPN may also increase long-term morbidity in cancer survivors (170, 171). Despite the prevalence of CIPN in cancer patients, there is no conclusive treatment plan for either prophylaxis or symptom management. Gabapentin, Lamotrigine, Amitriptyline and NSAIDs have proven ineffective leaving many patients with opioids as the only option for pain mitigation (172). At the time of this review, there is a paucity of peer-reviewed literature examining the efficacy of local steroid treatment for CIPN. Modest success to reduce neuritis pain has been achieved using systemic lidocaine administration (pregabalin dosed at 450 mg/day) following treatment for metastatic cancer of the ilium (173).

#### 4.5.4 Idiopathic neuropathies

Brachial neuritis, also known as Parsonage-Turner syndrome, is a rare disease of idiopathic origin presenting as acute proximal upper extremity pain followed by weakness (174). Less commonly, there is a hereditary form, hereditary neuralgic amyotrophy, caused by an autosomal dominant mutation in the septin 9 gene of chromosome 17 responsible for cytoskeleton formation (175). The ill-understood etiology of this disease is likely the cause of insufficient research examining treatment options. Nevertheless, corticosteroids have been indicated as a possible treatment modality. A review from 1960 to 2009 identified no randomized controlled trials of treatment for brachial neuritis but did recommend early initiation oral prednisone therapy (within the first month after symptom presentation) to increase recovery speed (176). A subsequent study in 2016 recommended a similar treatment of an immediate “short trial of high-dose oral corticosteroids” (prednisolone) in agonized patients as part of a multimodal recovery plan (177). A 2018 case study of a 6-month-old child suffering from brachial neuritis following an upper respiratory tract infection reported a full recovery by 16 months after treatment with prednisolone and physical therapy (178). The studies above suggest that corticosteroid therapy may be effective for brachial neuritis when early treatment is provided. Although steroids have been used to treat brachial neuritis, there still lacks controlled studies to assess the efficacy of this therapy (176, 177).

### 4.6 Safety and adverse effects

The broad therapeutic spectrum of steroids makes them efficient treatment for acute and chronic inflammatory diseases (5). Unfortunately, this broad spectrum carries over into side effects of physiologic signaling disruption. It is notable, however, that locally injected steroids pose less risk of adverse effects than oral steroids (179), and the research cited below regards long-term use of high dose oral steroids.

In the perioperative setting, side effects include sodium and water retention, increased risk of peptic ulceration, hypokalemia, increased infection rates, and adrenal crisis (132, 180). The mechanisms involved in long-term side effects are poorly understood, but conditions include osteoporosis leading to fractures, wound repair inhibition, osteonecrosis, development of cushingoid features, hypothalamic–pituitary–adrenal (HPA) axis suppression, hyperglycemia, dermatologic, ophthalmologic, and cardiovascular effects (1, 181–184). Systematic reviews have concluded that these adverse effects are dose and duration dependent (1, 182). For example, one study of 1,066 patients found an elevated occurrence of adverse effects beyond certain thresholds. Specifically, prednisone dosages greater than 7.5 mg per day could cause glaucoma and hypertension while dosages greater than 5 mg per day could cause weight gain (185).

Steroid induced osteoporosis may additionally result from such treatment as 6 mg of prednisone per day for only 6 months has been noted to lead to bone loss and fractures (186). This statement is supported by another study which examined glucocorticoid-induced osteopenia and found that 53% of patients receiving a cumulative prednisone dose of greater than 30 mg had fractures (187).

Although most dermatologic effects are benign, impaired wound healing is consequential. However, steroid-inhibited wound repair is uncertain and should be further examined (188). While some studies have associated steroid use for rheumatoid arthritis with increased risks of infection (189, 190), a meta-analysis of 38 studies concluded that there was no difference in postoperative infection rates between surgical patients treated with dexamethasone as compared to no treatment or placebo (188).

As for peptic ulcer disease, a retrospective study of 1,415 patients found a two-fold increased risk for peptic ulcer disease in patients taking corticosteroids and relative risk of 4.4 in patients simultaneously taking NSAIDs and corticosteroids (191). Corticosteroid effects on the HPA axis are clearer; corticosteroids suppress hypothalamic corticotropin-releasing hormone, anterior pituitary adrenocorticotropic hormone, and adrenal cortex cortisol (184). One study found 5 mg of prednisolone per day for 1 month duration increases risk of HPA axis suppression while another study reported that 100% of patients undergoing long-term glucocorticoid use experience adrenal insufficiency (132, 192).

The hyperglycemic effects of steroids can eventually lead to diabetes mellitus which alone has an abundance of long-term deleterious effects. One report found a strong correlation between accumulated prednisone and the development of diabetes mellitus (193).

Aside from detrimental long-term adverse effects, short-term high doses can result in acute psychiatric symptoms. In children, high-dose corticosteroid toxicity has occasionally resulted in acute psychosis (184, 194).

Furthermore, the route of administration of steroids has also been implicated in adverse events. Case reports have described iatrogenic nerve injury following steroid injection for carpal tunnel syndrome (195). Additionally, repetitive steroid injections are implicated in worse postoperative complications in carpal tunnel release as well as tendon and fascial ruptures (4, 196). Nerve and tendon injuries could be mitigated through careful, ultrasound-guided injection; however, increased pain and fascial tears are primarily an unavoidable result of needle insertion.

### 4.7 Future directions

While there is evidence that steroids decrease inflammation and improve nerve regeneration/functionality, there is still a gap in the literature as to whether they have a defined therapeutic use in the treatment of peripheral nerve injury. Furthermore, peripheral nerve injury treatment still lacks a comprehensive regimen for regaining complete functionality and addressing nerve dysesthesia (197, 198). We recommend future studies to address these topics, in addition to elucidating the role of local steroids in multimodal pain treatment.

Some future avenues of steroid use in peripheral nerve injury to explore include radial tunnel syndrome, ulnar neuropathy, meralgia paresthetica, brachial neuritis, CIDP, diabetic neuropathy, and CIPN. Future data is needed to assist in timing of use, length of use, dosage, defining when to transition from nonoperative treatment to surgical intervention, as well as discussion of when injections prior to surgery play a role in surgical outcomes (199).

Additionally, we recognize the importance of leprosy neuropathy among treatable peripheral neuropathies, particularly in light of recent observations in Florida, United States, where a previously undetected endemic was identified. Given the significance of this condition, we suggest that leprosy neuropathy should be the subject of a separate, dedicated systematic review to further explore its implications and treatment options.

## 5 Conclusion

This review highlights the lack of cohesive literature regarding the use of corticosteroids in various peripheral nerve disorders. Amid the uncertainty, promising results have been obtained on the use of steroids in addressing peripheral nerve injury and assisting nerve regeneration (4, 29, 39, 50, 101, 176, 196–198, 200–203). Given the wide range of clinical indications for steroids, few applications have been studied with sufficient depth. Due to anti-inflammatory and regenerative effects on peripheral nerves, steroids may be a beneficial adjunct in multi-modal pain treatment to improve pain after peripheral nerve surgery, useful in compression neuropathy, and a useful arm of the management of non-compression neuropathy. We suggest more focused investigation into the mechanisms of corticosteroids as potentially favorable adjuvants and clinical trials in the conditions for which they may provide improved treatment.

## Data Availability

The original contributions presented in the study are included in the article/supplementary material, further inquiries can be directed to the corresponding author.
